# Supplementary material for: Choroidalyzer: An Open-Source, End-to-End Pipeline for Choroidal Analysis in Optical Coherence Tomography
Source: Invest Ophthalmol Vis Sci. 2024 Jun 4;65(6):6. doi: 10.1167/iovs.65.6.6 (PMC11156207; doi:10.1167/iovs.65.6.6)
Supplement: Supplement 1 [file iovs-65-6-6_s001.pdf]

Supplementary Material

1. Population statistics across training, validation and test sets

	Training	Validation	Testing	External test	Total
Subjects	122	28	37	46	233
Male/Female	64 / 57	12 / 16	16 / 21	24 / 22	116 / 116
Control/Case	76 / 46	16 / 12	20 / 17	0 / 46	112 / 121
Right/Left eyes	117 / 107	27 / 23	37 / 28	46 / 0	227 / 158
Standard/FLEX/DRI Triton Plus	88 / 14 / 20	24 / 2 / 2	29 / 6 / 2	46 / 0 / 0	187 / 22 / 24
Heidelberg/Topcon	102 / 20	26 / 2	35 / 2	46 / 0	209 / 24
Age (mean (SD))	40.7 (14.2)	42.5 (11.9)	44.5 (13.4)	47.5 (12.3)	42.9 (13.4)
Cohort					
OCTANE	0	0	0	46	46
Diurnal Variation	12	4	4	0	20
Normative	1	0	0	0	1
i-Test	13	2	6	0	21
Prevent Dementia	76	20	25	0	121
GCU Topcon	20	2	2	0	24
B-scans					
Standard/Flex/DRI Triton Plus	582 / 2,281 / 1,281	136 / 190 / 140	137 / 462 / 157	168 / 0 / 0	1,023 / 2,933 / 1,578
Heidelberg/TopCon	2,863 / 1,281	326 / 140	599 / 157	168 / 0	3,956 / 1,578
Horizontal/Vertical scans	462 / 461	90 / 82	95 / 95	168 / 0	816 / 638
Volume/Radial/Peripapilary scans	2,161 / 1,060 / 39	178 / 116 / 15	434 / 131 / 12	0 / 0 / 0	2,773 1,307 / 0
Total B-scans	4,183	481	768	168	5,600

Table S1 Overview of population and image characteristics of the internal training, validation and test sets, and also the external test set. Note that one participant's sex from the Topcon cohort was not recorded. SD: Standard Deviation.

2. Full comparison metrics with methods and manual graders

Comparison	Region		Vessel		Thickness				Area				Vascular Index			
	AUC	Dice	AUC	Dice	pearson	spearman	ICC	MAE (μm)	pearson	spearman	ICC	MAE (mm^2)	pearson	spearman	ICC	MAE
M1 vs. M2	0.9639	0.9474	0.8891	0.7699	0.9503	0.9521	0.9783	17.8833	0.9516	0.9248	0.9751	0.1096	0.8074	0.6857	0.8172	0.0618
M1																
Choroidalyzer	**0.9964**	**0.9242**	**0.9896**	0.7410	0.9322	**0.9490**	**0.9761**	27.2167	**0.9211**	**0.8872**	0.9570	**0.1598**	**0.7668**	**0.8406**	**0.7265**	**0.0555**
SOTA	0.9370	0.9227	0.9271	**0.7714**	**0.9437**	0.9378	0.9676	**25.8500**	0.9198	0.8692	**0.9589**	0.1631	0.7150	0.6857	0.7157	0.1901
M2																
Choroidalyzer	**0.9993**	**0.9507**	**0.9933**	**0.7927**	0.9746	0.9838	**0.9984**	14.7333	0.9896	**0.9865**	0.9942	**0.0702**	0.5640	**0.6361**	**0.7960**	**0.0506**
SOTA	0.9175	0.9439	0.9175	0.7770	**0.9914**	**0.9894**	0.9927	**14.0000**	**0.9897**	0.9774	**0.9948**	0.0770	**0.6663**	0.5353	0.7047	0.1464

Table S2 Full comparison metrics between Choroidalyzer, two manual graders M1 and M2, and state of the art region and vessel segmentation methods DeepGPET and Niblack. AUC: Area under the Receiver Operating Characteristic Curve. MAE: Mean Absolute Error. ICC: Intra-Class Correlation.

3. Analysis effects of fovea location error on downstream metrics

Choroidalyzer measured the fovea column coordinate with a median absolute error of 3 pixels in both the internal and external test sets. We tested the effect of perturbing the fovea column on choroidal metrics by comparing fovea-centred metrics and metrics derived after the fovea column was randomly perturbed using a discretised uniform distribution $\sim U(-6, 6\}$ (excluding 0). 50 simulations were run on approximately 10% of the dataset (495 OCT B-scans), selected at random to represent eye type and location on the macula (see supplementary Table S3 for a description on the image statistics of this random sample).

All metrics had excellent Pearson correlation ($r > 0.99$, p<0.00001, supplementary Fig. S1). Scatterplots of metrics for the poorest performing simulation according to absolute error across all metrics (supplementary Fig. S2) shows excellent agreement with the

Eyes (Number of scans)	OD	OS		Total
	42 (263)	31 (232)		73 (495)
Location	H-line/V-line	Ppole/Radial		
	85/64	217/129		495
Device	OCT1 (Heidelberg)	OCT2 (Heidelberg)	DRI Triton Plus (Topcon)	
	113	225	157	495

Table S3 Image statistics of the random sample of 495 OCT B-scans used to understand the effects of random perturbations of the fovea coordinate.

identity line, with limits of agreement in the Bland-Altman plots well within acceptable bounds for all metrics[48;49]. Thus, the fovea column quantitative error observed from Choroidalyzer does not significantly impact the choroidal metrics.

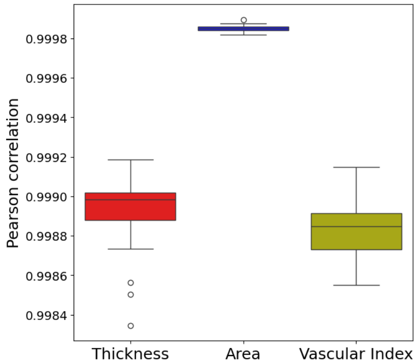

Figure S1 Distribution of Pearson correlation coefficients for each choroidal metric when perturbing the fovea coordinate column. Note the scale of the y-axis, even the lowest correlation we observed was > 0.99.

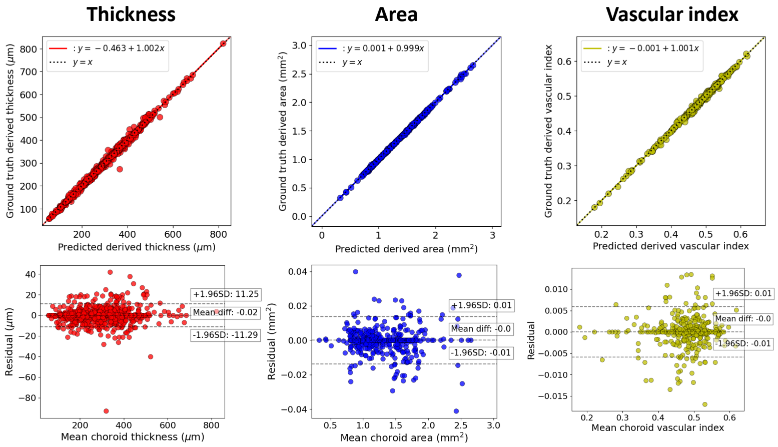

Figure S2 Correlation and Bland-Altman plots for choroidal metrics for the poorest performing simulation of perturbing the fovea column coordinate on a random 10% subsample of the dataset.

4. Comparison of MMCQ and Niblack segmentation methods

Fig. S3 shows some qualitative examples between the vessel segmentations produced by MMCQ and Niblack for exemplar B-scans from all imaging devices used in this study. Table S4 shows the results of comparing MMCQ and Niblack with the 20 OCT B-scans from the external test set which were manually segmented by two experienced graders (I.M. and J.B.). We found that both approaches performed similarly when compared to the manual grader. We did observe a large mean absolute error in CVI when comparing the two approaches directly. We believe this is due to Niblack having a tendency to oversegment the choroid, such that it is able to segment all vessels in the choroid at the cost of segmenting parts of the interstitial space. MMCQ instead attempts to preserve vessel fidelity by not segmenting the interstitial space — at the cost of rejecting ambiguous pixels which either represent vessel walls or interstitial space.

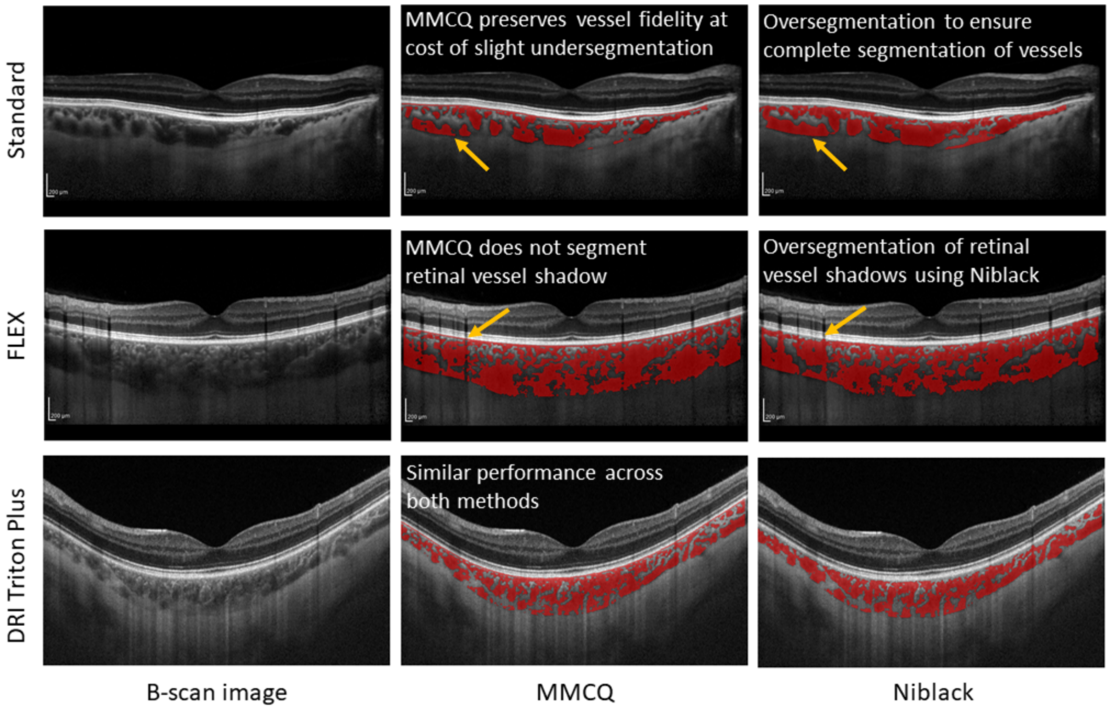

Figure S3 Examples of MMCQ (centre column) and Niblack (right-hand column) segmenting exemplar OCT B-scans from each imaging device, the Heidelberg Standard module, the Heidelberg FLEX module, and the Topcon DRI Trition plus.

Comparison	CVI			Vessel segmentation	
	Pearson	Spearman	MAE	Dice	AUC
Niblack vs. Manual (Avg)	0.560916	0.663158	**0.083508**	0.746164	0.820954
MMCQ vs. Manual (Avg)	**0.679932**	0.627068	0.088144	**0.819151**	**0.903037**
Niblack vs. MMCQ	0.699646	0.781955	0.159811	0.777819	0.948477

Table S4 Vessel segmentation and choroid-derived CVI metrics between average manual segmentation, Niblack thresholding algorithm and MMCQ. CVI: Choroid vascular index.

5. Analysis effects of Choroid- and Image-aligned regions of interest

We investigated the effects that different regions of interest aligned with either image axis or choroid axis had on choroid measurements. Our initial hypothesis was that B-scans of highly myopic eyes could skew the choroid off-centre from the image axis, which could have a noticeable impact on choroidal measurements. We conducted two forms of analysis to test this hypothesis, in both cases comparing measurements made according to the horizontal image axis, and the choroid axes.

First, we used the same random sample of OCT B-scans used in supplementary section 3 (Table S3). We compared choroidal measures for different axis alignment (Fig. S4) and found that choroidal thickness differed significantly between different alignments as the size of the choroid increased, while area and CVI remained highly reproducible. We suspect this is because choroid thickness is

a one-dimensional straight line distance measure, which can be highly susceptible to changes in pixel length-scale. This is emphasised in OCT B-scans as the axial and lateral resolution are different (approximately 4:1) in Heidelberg and Topcon imaging devices.

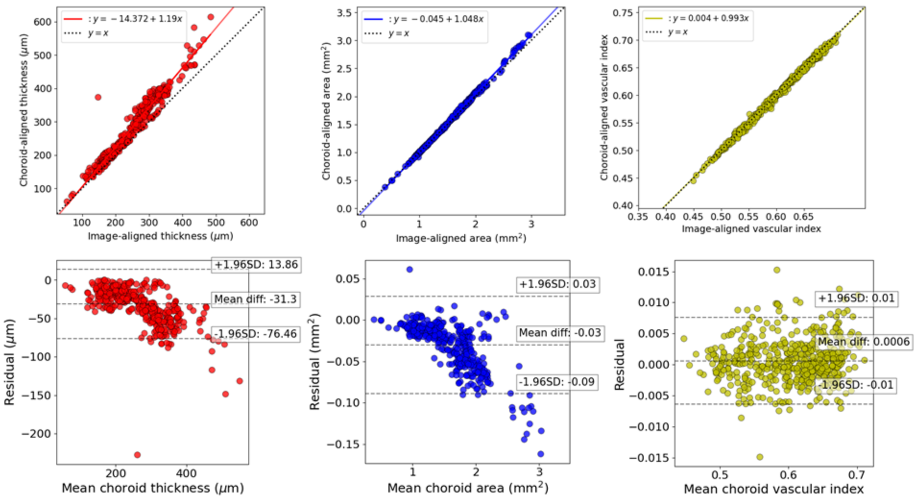

Figure S4 Correlation and bland-Altman plots of measuring thickness, area and CVI using the choroid axis (x) and the image axis (y).

Secondly, we investigated any effect that myopia might have on these measurements. We selected three individuals from the GCU Topcon cohort: one highly hypermetropic (6.375 Spherical Equivalent Dioptres (D)), one emmetropic (0.75D) and one highly myopic (-7.5D) individual. A random B-scan was segmented and thickness (at macular locations described in the main paper, i.e. subfoveal and 2mm temporal and nasal to the fovea), area and CVI was measured. Table S5 shows the measurements for these three OCT B-scans, measuring both aligned to the choroid axis and the image axis. Fig. S5 shows the three OCT B-scans with choroidal thickness and area measurements annotated.

Individual	Thickness, (N, F, T) (microns)			Area (mm2)			Choroid vascular index (CVI)		
	Choroid	Image	Largest residual	Choroid	Image	Residual	Choroid	Image	Residual
Hypermetropic (6.375D)	(541, 762, 442)	(452, 686, 355)	89 microns (N)	3.481	3.497	0.015	0.731	0.728	0.003
Emmetropic (0.75D)	(248, 377, 427)	(232, 337, 318)	109 microns (T)	1.928	1.881	0.046	0.607	0.606	0.001
Myopic (-7.5D)	(212, 391, 474))	(198, 342, 368)	106 microns (T)	1.928	1.880	0.048	0.697	0.692	0.005

Table S5 Comparisons of thickness, area and vascular index measured aligned with the choroid axis and with the image axis. For each choroid measure, the values for each type of alignment are shown, as well as the absolute value residual. For thickness, we selected the largest residual across the macular location for readability. N, nasal; F, subfoveal; T, temporal.

In Table S5 we see that the significant errors lie within the choroid thickness measurements, and there is no discernible difference in error between the three individuals, regardless of their degree of myopathy. In Fig. S5 we observe the choroidal curvature in all three images (in particular, temporal to the fovea). The degree of this curvature roughly corresponds to the degree of variation of the thickness measurement (green vs. cyan lines) and region of interest definition (shaded green vs additional blue shaded region).

Our results appear to generally contradict our initial hypothesis that high myopia could affect the choroidal measurements. In fact, the primary cause for large differences between choroid-aligned and image-aligned measurements are the extent of deviation of the choroid axis from the image axis, the size of the choroid, and not the extent of myopathy of the eye. The factors which likely contribute to this curvature are imager experience and patient concentration.

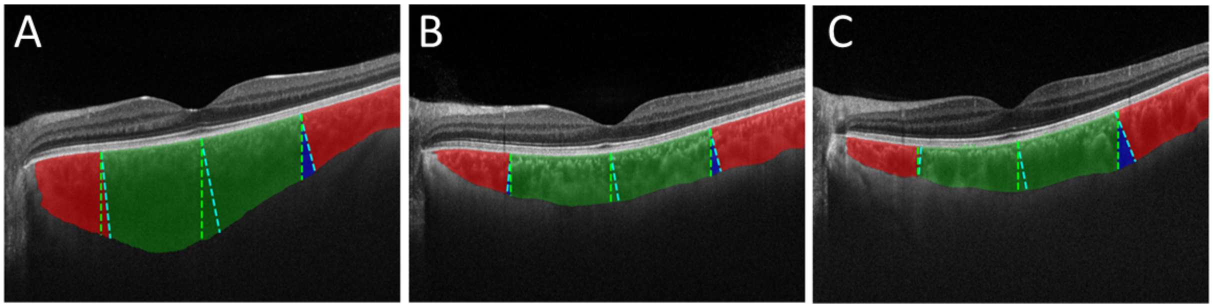

Figure S5 Choroid thickness and area for hypermetropic (A), emmetropic (B) and myopic (C) choroids. Thickness shown as straight lines with green representing image-aligned measurement and cyan as choroid-aligned measurement. Area in shaded green shows overlap between image- and choroid-aligned regions of interest, with blue as regions which were only Choroid-aligned. Shaded red are regions of the choroid not measured within any region of interest.
